# Microdrilling of Through-Holes in Flexible Printed Circuits Using Picosecond Ultrashort Pulse Laser

**DOI:** 10.3390/polym10121390

**Published:** 2018-12-14

**Authors:** Wanqin Zhao, Lingzhi Wang

**Affiliations:** 1 School of Materials Engineering, Shanghai University of Engineering Science, Shanghai 201620, China; 18321261951wang@gmail.com; 2 Shanghai Collaborative Innovation Center of Laser Advanced Manufacturing Technology, Shanghai 201620, China

**Keywords:** microdrilling, high-density, through-holes, picosecond ultrashort pulse laser, FPCs

## Abstract

High density and high quality interconnects are necessary for the preparation of miniaturized and lightweight electronic products. Therefore, small-diameter and high-density through-holes in FPCs (Flexible Printed Circuits) are required. However, the current processing methods cannot further decrease the diameters and improve the quality of through-holes. Comparatively, ultrashort pulse laser is a good choice. In this paper, the processing technology for the microdrilling of through-holes in FPCs using a 10 ps pulse laser was systematically studied. The effects of laser parameters, including the wavelength, energy, pulses and polarization, on the drilling of through-holes were investigated. The various processing parameters were optimized and the plausible reasons were discussed. Finally, the desired small-diameter and high-density through-holes in FPCs were obtained. The experimental results showed that, through-holes with diameters of less than 10 µm and inlet interconnection pitches of 0~2 µm could be successfully drilled in FPCs using ultrashort pulse laser.

## 1. Introduction

Flexible printed circuits (FPCs) are composite laminated materials that are mainly composed of copper (Cu) and polyimide (PI). Cu foil is used as the conductive metal material, while the typical organic polymer PI serves as the dielectric or insulating layer due to its advantages of good dielectric property, thermal stability, excellent mechanical properties, plasticity and less fragility [1]. Based on their structural and material characteristics, FPCs display many desirable properties, such as high reliability, flexibility, light weight and thinness. FPCs have been applied in various fields including the 3C products of the computer, communication and consumer electronics. The future trend of electronic products is light, thin, short and small. Therefore, it is necessary to process the FPCs with high density and quality interconnects. Microhole drilling is the main processing technology to obtain the interconnects in FPCs. There are strict requirements for the size and quality of the microholes drilled in FPCs, which directly determine the density and performance of interconnects.

For drilling through-holes in FPCs, CO_2_ lasers with wavelengths in the range of 9.2–10.6 μm and solid-state UV nanosecond lasers with wavelengths in the range of 266–355 nm are the most commonly used at present [2,3,4]. However, the processing mechanism of CO_2_ lasers involves heating ablation which leads to some obvious defects, such as excess carbide formation and collapse between layers [5]. Moreover, there is very low absorption ratio for Cu at the near-infrared wavelength, which makes the drilling unsuitable directly using CO_2_ laser in FPCs. Therefore, a coating metal is required on the surface of Cu resulting in complex processing [6], or the Cu layer is stripped using other lasers and then another layer is processed using CO_2_ lasers [7]. Moreover, the obtained hole diameters using CO_2_ lasers are approximately 75~150 μm which could not be further decreased [8]. Regarding the UV nanosecond lasers, the ablation mechanism involves two aspects, the photochemical effect and the photothermal effect [9,10]. In the photochemical effect, the chemical synthesis is broken down by photons when the photon energy is higher than the bonding energy of molecules. Satisfactorily, it does not result in the ablation defects. In the photothermal effect, thermal ablation is involved due to the higher laser energy, which makes the materials melt. Therefore, the effect causes obvious molten spatters around the periphery of holes and recast layers on the side-wall surfaces. Moreover, the diameter of the hole ablated using UV nanosecond lasers could only reach up to 25 μm [8]. In other words, the problems on microdrilling of through-holes in FPCs mainly include decreasing the hole diameter and enhancing the hole quality in order to realize high-density and high-quality interconnects.

Ultrashort pulse lasers are lasers having pulse durations in the picosecond to femtosecond range [11]. The advantages of ultrashort pulse laser processing include reducing or even eliminating the thermal effects, namely the cold ablation [12,13], so that high quality holes could be drilled. Moreover, the technique is not affected by the diffraction limit or the material properties. Thus, micro– and nana–holes can be obtained and almost all materials can be directly ablated [14,15]. Therefore, based on these advantages, ultrashort pulse lasers have become a popular choice for directly drilling high quality micro through-holes in multi-layered composite materials, such as FPCs. For example, Ohnishi et al. reported that microholes with diameters of less than 10 µm had been ablated in PI using ultrashort pulse lasers with the pulse durations of 70 fs~2.2 ps. It was reported that the shorter pulse duration was better for forming a microhole with a high degree of precision [16]. Zhu et al. investigated drilling holes in Cu foil with the thickness of less than 50 µm using a 60 fs laser [17]. Nevertheless, whether the material was PI or Cu foil, the spatters around the periphery of the microholes still existed even when the pulse duration was reduced to a few tens of femtoseconds. In fact, more precise drilling cannot be expected by simply lowering the pulse duration in such cold ablation techniques. In addition, serious non-linear optical effects, such as the self-focusing of the beam [18] and the conical emission [19], would be stimulated and affect the ablation quality. Moreover, picosecond lasers with the pulse durations of less than 10 ps, which also belong to the ultrashort pulse laser category, can achieve high quality ablation by optimizing the processing technology. New generation high power picosecond lasers are now available on the market for the ablation of blind- and through-holes with high quality and throughput production in printed circuit boards made up of Cu and FR4 [20]. However, systematic research on the microdrilling of through-holes in composite laminated material FPCs using ultrashort pulse laser is lacking. In this paper, a series of experiments was performed on microdrilling of through-holes in FPCs using picosecond ultrashort pulse laser with diverse parameters including the laser wavelength, energy, pulse and polarization. The effects of the processing parameters on the dimensions and morphologies of through-holes in FPCs were studied. Moreover, small-diameter and high-density through-holes in FPCs were processed to evaluate the feasibility of this method for practical commercial applications.

## 2. Materials and Methods

The laser utilized for irradiation in this paper was a neodymium-vanadate laser (High Q IC-1500 Nd:VAN; High Q Laser Innovation GMBH, Rankweil, Austria) with the pulse duration of 10 ps, repetition rate of 1 kHz and wavelengths of 355, 532 and 1064 nm. Multiple optical lenses with the focal lengths of 25~150 mm were used to focus the laser beam on the surface of samples. The experiments were performed in air atmosphere. After the experiments, the size and morphology of through-holes were measured mainly using scanning electron microscopy (SEM, Hitachi Ltd., Tokyo, Japan). Furthermore, in order to obtain the cross-section morphology of through-holes, the samples with large area through-holes were inlaid using transparent gel and then were split using sandpaper and polishing agents. The absorptions of Cu and PI under various wavelengths were measured using ultraviolet and visible light spectrophotometer (UVLS, Shimadzu Corporation, Tokyo, Japan). 

**Figure 1:**
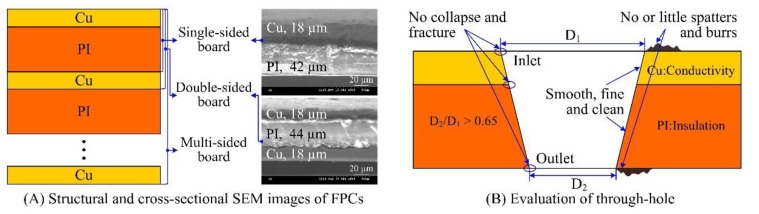
Geometry of Flexible Printed Circuits (FPCs) and the evaluation of through-hole in FPCs.

The FPC samples composed of Cu and PI mainly include single-, double- and multi-sided boards, as shown in Figure 1A. Moreover, the Cu and PI layers had different thicknesses. For example, the single-sided board was composed of Cu with the thickness of 18 μm and PI with the thickness of 42 μm, while the double-sided board had 18 μm Cu and 44 μm PI, as shown in Figure 1A. Moreover, in the study on high-density through-hole ablation, a 30 μm PI layer on the surface of 46 μm Cu was used instead.

The evaluations of the through-holes drilled in FPCs are shown in Figure 1B. For the dimensions, the ratio between the outlet diameter and inlet diameter should be larger than 0.65 to ensure proper taper of the through-holes. For the morphology, there should be few or no molten spatters and burrs around the periphery of the through-holes. As seen from Figure 1, there was no collapse and fracture at the copper-dielectric interface. There was smooth and fine side-wall morphology without the recast layer on the side-wall surface. Especially, there was no Cu at the non-conducting region and no PI at the conducting area based on the conductivity of Cu and the insulation of PI. That is, there was no other material with different electrical property on the surface of a layer to retain the independent functions of the different material layers.

## 3. Results and Discussion

When a metallic material is irradiated using ultrashort pulse laser, based on the inverse bremsstrahlung, laser energy is absorbed by the free electrons in the skin depth [21]. At this point, the electrons heat up then, the electrons get redistributed and reach the thermal equilibrium state within the time scale of hundreds of femtoseconds to several picoseconds [22]. Hereafter, the energy absorbed is transferred from the electrons to the lattices. Moreover, the transfer time is mainly determined by the electron-phonon coupling constant and is generally 1~100 ps, which is longer than the time required for the electrons to reach thermal equilibrium [12]. As a result, only a small part of the energy is converted into the heat source, thus realizing the precise machining ability of ultra-short pulse laser [14]. Different from the metallic materials containing large amounts of free electrons, the absorption mechanism of laser energy in dielectric materials is entirely different. When a dielectric material is irradiated using ultrashort pulse laser, photo ionization and avalanche ionization are stimulated in the processed area and a large number of free electrons are generated. Furthermore, the thin layer on the surface of dielectric material is transformed into plasmas with metal-like properties [23]. Subsequently, the photons are absorbed by the electrons in the dielectric material based on the interaction between the laser and plasmas. The phenomena of material melting, peeling, expansion and boiling would occur, causing the material to be eliminated [24]. In conclusion, high precision micromachining in both metal and dielectric materials can be expected using ultrashort pulse laser. Therefore, the processing for microdrilling of through-holes in the composite laminated material FPCs composed of Cu and PI was studied comprehensively, as described in the following sections.

### 3.1. Processing Technology for Microdrilling of Through-Holes

#### 3.1.1. Laser Wavelength Effect

**Figure 2:**
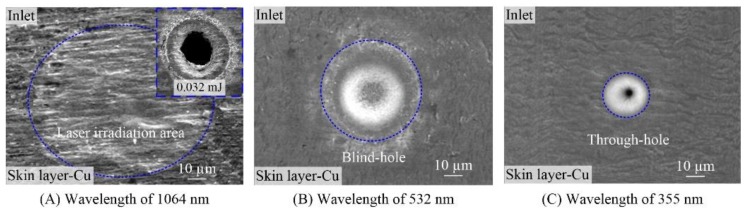
Effect of laser wavelengths on the holes in double-sided board (Energy of 0.005 mJ, 50 pulses).

Figure 2 presents the SEM images of the through-holes in FPCs ablated with the laser wavelengths of 1064, 532 and 355 nm. It can be seen from Figure 2 that the ablation area changes from chemical and structural variations to the blind-hole, then to the through-hole, when the laser wavelengths varied from 1064 nm to 532 nm then to 355 nm with the other processing parameters the same. In other words, the utilization rate of the laser energy was highest for the shortest laser wavelength. Besides, the shorter the wavelength, the smaller the hole diameters. Therefore, the short wavelength had a great advantage in the small-diameter hole drilling. Furthermore, based on the relationship between the focal spot *ω*_0_ (at 1/e amplitude) and the laser wavelength *λ*, as shown in Equation (1):

(1) $$\omega_{0}=\frac{M^{2\;}f\lambda }{\pi \omega_{s}}$$

where *ω*_s_ is the initial beam radius and is about 2.4, 1.3 or 0.78 mm for wavelengths of 1064, 532 or 355 nm in Figure 2, respectively. *λ* is the laser wavelength. *f* is the focal length of the optical lens and equals to 150 mm for the holes ablated in Figure 2. *M*^2^ is the beam quality factor and is 1.33 [25]. Then, *ω*_0_ can be calculated and is approximately 28.17, 26 and 28.91 µm for wavelengths of 1064, 532 and 355 nm in Figure 2, respectively. That is, there are almost similar focal spots for three different laser wavelengths. In other words, the focal spot radius has little effect on the holes diameters. Thus, other parameters related to the laser wavelength, such as the material absorptivity and threshold, have a great influence on the hole diameter. In addition, obvious molten spatters and rough side-wall were observed for the through-hole drilled with the wavelength of 1064 nm as shown in the upper-right corner of Figure 2A. The results showed that the 1064 nm wavelength was unsuitable for hole drilling in FPCs. Comparatively, few molten spatters and fine side-wall were observed for the holes ablated with the wavelengths of 532 and 355 nm, as shown in Figure 2B,C. In conclusion, visible light, especially ultraviolet light, was superior to near-infrared light in the microdrilling of small-diameter and high-quality through-holes.

**Figure 3:**
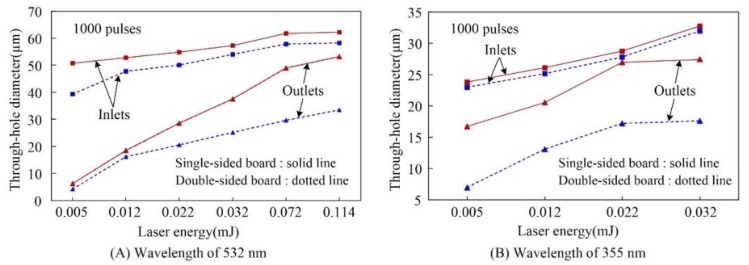
Hole diameter versus energy with different wavelengths.

The laser wavelength also affected the saturation state of the inlets and outlets. As shown in Figure 3, the diameters of both inlets and outlets initially increased and then reached a saturation state along with the increase in laser energy. This trend was consistent with the results of other studies on the effect of laser energy on hole diameter [26]. Furthermore, plasma in the inlet and the scattering of the beam within the plasma are the main causes inducing the hole diameter increase. Meanwhile, it must be pointed out that the effect would reach the limitation eventually. Take as an example, when the plasma density reaches the critical value, the beam will be reflected [27]. At this point, the plasma, as the energy source, will reach saturation and the holes diameter also reaches saturation. In addition, the saturation energies were different for the inlets and outlets. For example, when the laser wavelength was 532 nm with the energy of 0.072 mJ, as shown in Figure 3A, the inlet diameters reached the maximum value while the outlet diameters kept increasing gradually. Moreover, the inlet diameters increased gradually and the outlet diameters reached saturation when the laser wavelength was 355 nm with the energy of 0.022 mJ, as shown in Figure 3B.

**Figure 4:**
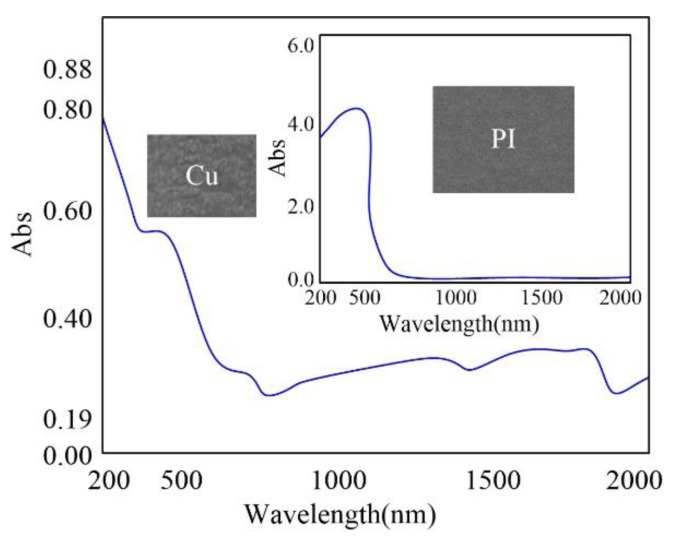
Absorptions of FPC materials.

For the inlet and outlet diameters, based on a large number of experiments, it was found that there are much more times for the inlets diameters saturation with the outlets diameters still increasing gradually for the wavelength of 532 nm and it is reverse for the wavelength of 355 nm. This phenomenon could be ascribed to the different absorptivities of the FPC materials for different laser wavelengths, as shown in Figure 4. Specifically, the absorptions of Cu and PI at the wavelengths of 355 and 532 nm were measured using UVLS. Then, the absorptivities of Cu and PI were calculated and were 73.70% and 94.90% at the wavelength of 355 nm and 59.26% and 68.53% at the wavelength of 532 nm, respectively. Comparatively, the absorptivity of PI was significantly higher than that of Cu for the wavelength of 355 nm. Therefore, for the laser ablation in single-sided board with outlet layer of PI and the wavelength of 355 nm, the outlet diameter was more likely to reach saturation. Furthermore, for the double-sided board with the inlet and outlet layers of Cu and the middle layer of PI, based on the high absorptivity of PI at the wavelength of 355 nm, the middle layer PI was more liable to reach saturation. Then, the saturated middle layer PI could be regarded as an aperture with the convergence effect for the outlet diameter. Therefore, the outlet diameter was more liable to be saturated. In other words, for the microdrilling of through-holes ablated with the laser wavelength of 355 nm, the outlet diameter was more liable to reach saturation for the single-sided board with the outlet layer of PI and the double-sided board with the outlet layer of Cu. On the other hand, the absorptivities of both Cu and PI are similar at the laser wavelength of 532 nm, as shown in Figure 4. Therefore, whether it was single- or double-sided board, the skin layer of Cu at the inlet was irradiated at first and thus, the inlet layer Cu was more liable to reach saturation.

#### 3.1.2. Laser Energy and Pulses Effects

**Figure 5:**
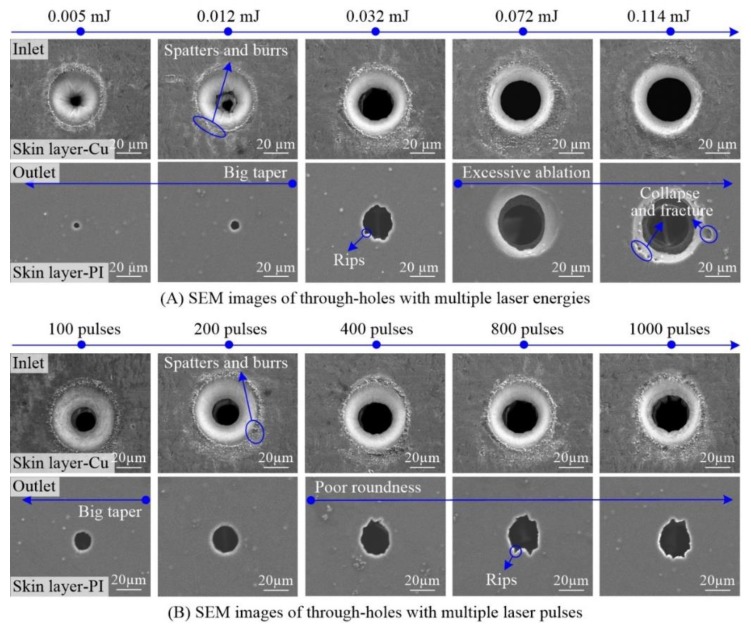
Effect of laser energy and pulses on the through-holes in single-sided board with the laser wavelength of 532 nm ((**A**) 500 pulses; (**B**) Energy of 0.032 mJ).

In order to ensure the effective ablation, the laser energy must be larger than the material ablation threshold. At the same time, for the pulsed laser machining, the laser energy is transmitted to the material through the pulses. In short, the laser energy and pulses have important effects on microhole drilling. Figure 5 shows the SEM images of through-holes in the single-sided board. Firstly, for the inlet morphology, it can be seen from Figure 5A that there were bits of molten spatters and burrs around the periphery of inlets and the molten materials firstly increased and then lessened with the increase in laser energy. Specifically, there were very few patters and burrs because of the weak ablation radiated by the low laser energy. As the laser energy increased, the molten spatters became more, such as the ablation with the energy of 0.032 mJ. On the other hand, as the laser energy continued to increase, the molten spatters did not continue to increase but reduced, such as the ablation with the energy of 0.114 mJ. The above-mentioned phenomenon had been reported and named the self-cleaning effect and the reason is ascribed to the influence of subsequent pulses on the surface spatters and burrs based on the Gaussian laser energy in space [28,29]. Secondly, for the outlet shown in Figure 5A, when the laser energy was less than 0.012 mJ, the outlet morphology was very good but the outlet diameters were too small, which made the taper of holes too big. The through-holes with big taper could not meet the requirement of through-hole drilling in FPCs. By increasing the laser energy, the outlet diameter obviously became larger. Especially, when the laser energy was more than 0.072 mJ, the inlet and outlet diameters were similar and the taper of through-holes was small, or even zero. However, very high laser energy caused the excessive ablation and even the collapse and fracture on the surface of outlet. Overall, the outlet quality was better but the roundness was poor with some rips for the through-hole ablation with the energy of 0.032 mJ.

Based on the un-excessive ablation of outlet with the energy of 0.032 mJ, through-holes were drilled with the energy of 0.032 mJ and multiple pulses in single-sided board. The SEM images of through-holes are shown in Figure 5B. It can be seen that there was little difference for the inlet diameters as the pulses increased from 100 to 1000. The roundness of inlets was very good and the side-wall morphology was very smooth and fine but there were some molten spatters and burrs around the periphery of the inlets. For outlets, the roundness was good with the pulses of less than 400 while it was poor with the pulses of more than 400 and some rips were observed. Moreover, the outlet diameter increased gradually when the pulses were less than 400. Thereafter, the outlet diameters remained almost consistent, indicating that the diameters reached the saturation state. In conclusion, both the inlet and outlet diameters reached the saturation state with the increase in laser energy and pulses. Furthermore, there were obvious spatters around the periphery of the inlets, while the excessive ablation with collapses and fractures and the poor roundness with some rips around the outlets for high energy and more pulses, respectively.

#### 3.1.3. Laser Polarization Effect

**Figure 6:**
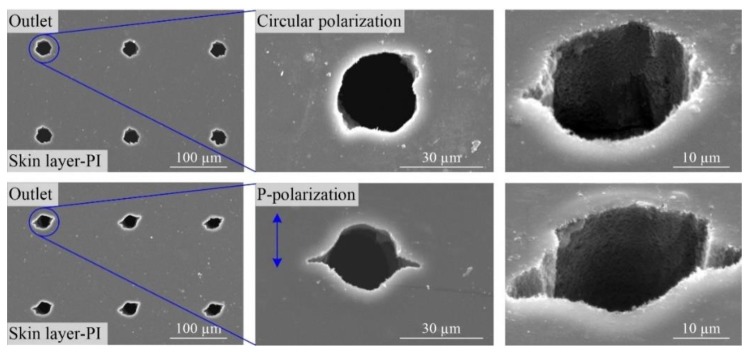
Scanning electron microscopy (SEM) images of through-holes with different laser polarizations in single-sided board with the laser wavelength of 532 nm (Energy of 0.007 mJ, 1000 pulses).

The above experiments illustrated that the rips around the outlets resulted in poor roundness of the holes. Although the rips could be avoided effectively by reducing the laser energy or pulses, the other characteristics of the outlets, such as the sizes and tapers, could not meet the requirement. In order to further optimize the roundness of outlet, the laser beam was adjusted to the circular polarization and the contrast experiments including the circular- and P-polarization were carried out with the higher laser energy and more pulses. The results are shown in Figure 6. First, there were always rips for the laser drilling with both circular polarization and P-polarization. Second, for the drilling with the P-polarization, there were always two rips in a straight line except the other ones. 

**Figure 7:**
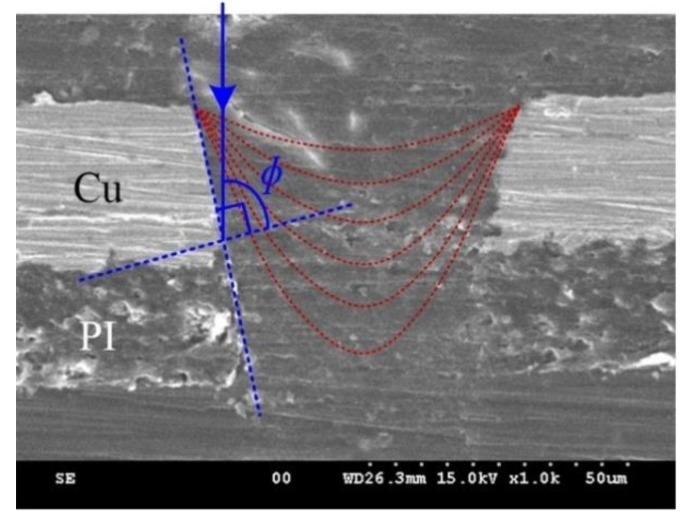
Schematic image of the incident angle between the direction of the incident beam and the surface normal.

The effect of laser polarization on the outlet has been reported. It was presented that there were two bulges located around the outlet and they were placed opposite to each other and oriented perpendicular to the direction of the polarization of the laser beam, which was confirmed by turning the direction of the polarization by 90° [30,31]. The reason is related to the different reflectivity in S- and P-polarization directions on the side-wall surface. They can be calculated using the Fresnel formulas [31,32]:

(2) $$R_{P}=\left(\frac{\sqrt{n^{2}-sin^{2}\emptyset }-n^{2}cos\emptyset }{\sqrt{n^{2}-sin^{2}\emptyset }+n^{2}cos\emptyset }\right){}^{2}$$

(3) $$R_{S}=\left(\frac{cos\emptyset -\sqrt{n^{2}-sin^{2}\emptyset }}{cos\emptyset +\sqrt{n^{2}-sin^{2}\emptyset }}\right){}^{2}$$

where *∅* is the incident angle between the direction of the incident beam and the surface normal, as shown in Figure 7, moreover, it changes from zero to about 90° (when the taper of the hole is zero) with the evolution of hole, *n* is the material refractive index.

### 3.2. Microdrilling of Through-Holes

Based on the above experiments on the processing technology of microdrilling of through-holes in FPCs using picosecond ultrashort pulse laser, high quality through-holes were processed. It is essential to prepare small-diameter and high-density through-holes in FPCs for the sake of practical commercial applications.

#### 3.2.1. Microdrilling of Small-Diameter Through-Holes 

**Figure 8:**
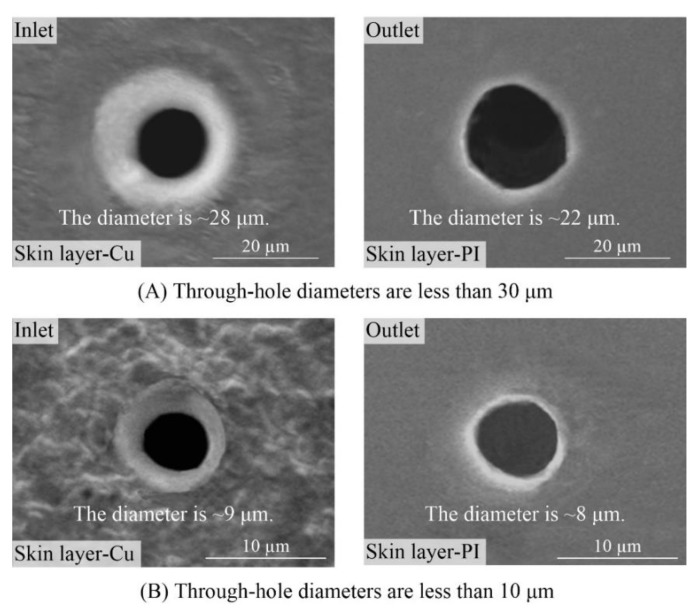
SEM images of through-holes in single-sided board with the laser wavelength of 355 nm ((**A**) Energy of 0.022 mJ, 50 pulses; (**B**) Energy of 0.005 mJ, 50 pulses).

Figure 8 shows the through-holes ablated using picosecond ultrashort pulse laser in single-sided board. The processing parameters were: laser wavelength of 355 nm, 50 pulses and energy of 0.022 mJ in Figure 8A and energy of 0.005 mJ in Figure 8B, respectively. For the through-holes dimensions, the inlet and outlet diameters were less than 30 or 10 μm for Figure 8A,B, respectively. For the morphology, the holes were round with well-defined edges, there were almost no molten spatters and burrs around the periphery of through-holes and the side-walls of through-holes were smooth, fine and clean. In other words, small-diameter and high-quality through-holes were successfully drilled in FPCs using picosecond ultrashort pulse laser.

#### 3.2.2. Microdrilling of High-Density Through-Holes

In order to explore the connectivity between the through-holes ablated using picosecond ultrashort pulse laser, large area through-hole groups with diversified pitches were processed in a single-sided board with 46 μm Cu and 30 μm PI. The main process parameters were: laser wavelength of 532 nm, energy of 0.063 mJ, repetition rate of 1 kHz, 1000 pulses, circular polarization beam and optical lens with the focus length of 25 mm. 

**Figure 9:**
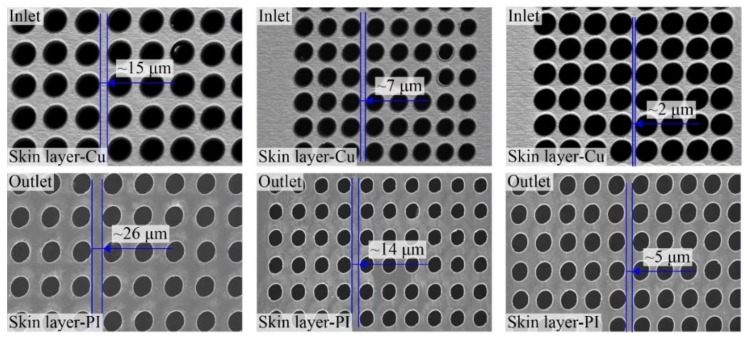
SEM images of through-holes with multiple inlet pitches in single-sided board.

Figure 9 shows the SEM images of through-holes in single-sided board with multiple inlet pitches of ~15 µm, ~7 µm and ~2 µm. The corresponding outlet pitches were ~26 µm, ~14 µm and ~5 µm, respectively. It can be seen that the consistency of the large-area through-holes ablated using picosecond ultrashort pulse laser was very good, which could fulfill the practical application. Besides, for the connection properties, the through-holes were completely independent even with the inlet pitch of only ~2 µm. The outlet pitches were much wider because the through-holes were all taper shaped, which also maintained the complete independence between the large-area through-holes.

**Figure 10:**
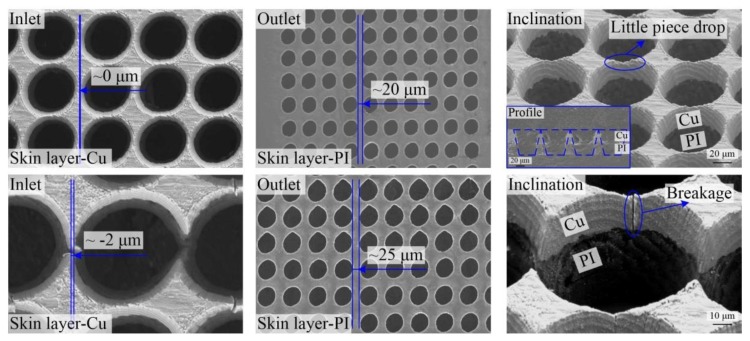
SEM images of through-holes with multiple inlet pitches in single-sided board.

With further decrease in the inlet pitch to zero as shown in Figure 10, the through-holes were still completely independent. Only one flaw appeared on a small piece of copper, as illustrated in the inclination image of Figure 10, with the inlet pitch of zero. Moreover, the outlets remained completely independent due to the taper of through-holes. Furthermore, when there was approximately 2 µm intersection between inlets, that is, the inlet pitch was minus 2 µm as shown in Figure 10, the outlet layer PI showed full independence. However, there were breakages on the inlet layer of Cu, which made the through-holes invalid.

**Figure 11:**
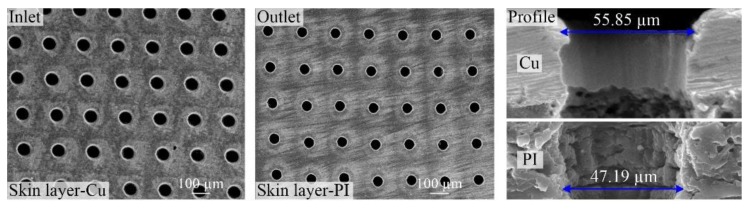
SEM images of through-holes and cross-sections in single-sided board.

For drilling high-density micro through-holes in FPCs, the independence between the through-holes needs to be ensured and also, the independent conductivity and insulation of the Cu layer and PI layer also need to be guaranteed. Therefore, the side-walls should remain clean for the through-holes processing. Next, the side-wall morphology of large-area through-holes drilled using picosecond ultrashort pulse laser was studied, as shown in Figure 11. It can be seen that there was residual free space on the side-wall surfaces for both the Cu and PI layers. Also, there was no collapse and fracture at the Cu layer, the PI layer and especially the copper-dielectric interface. In other words, high quality side-wall surfaces were achieved in the large-area through-holes in FPCs ablated using picosecond ultrashort pulse laser. Moreover, the independence of the different layers was realized, thus achieving their respective functions.

## 4. Conclusions

As electronic products are becoming increasingly miniaturized and light-weight, high density and high quality interconnects in FPCs are inevitable. In this paper, the microdrilling of small-diameter and high-density through-holes in FPCs using picosecond ultrashort pulse laser has been studied. First, the effects of laser parameters, including the wavelength, energy, pulses and polarization, on the drilling of through-holes drilling were investigated. It was found that visible light, especially ultraviolet light, was superior to near-infrared light for the drilling of small-diameter and high-quality through-holes. The use of excessively high energy and too many pulses resulted in ablation defects, such as spatters around the periphery of the inlets, collapses and fractures, poor roundness with some rips around the outlets and so forth. By optimizing the process conditions, the desired small-diameter and high-density through-holes in FPCs were obtained. Experimental results showed that through-holes with diameters less than 10 µm and inlets interconnection pitches of 0~2 µm could be achieved. Therefore, high density and high quality interconnects can be realized in FPCs using ultrashort pulse laser.
